# Identification of miRNAs associated with sexual maturity in chicken ovary by Illumina small RNA deep sequencing

**DOI:** 10.1186/1471-2164-14-352

**Published:** 2013-05-26

**Authors:** Li Kang, Xinxing Cui, Yujie Zhang, Chunhong Yang, Yunliang Jiang

**Affiliations:** 1 Shandong Provincial Key Laboratory of Animal Biotechnology and Disease Control and Prevention, College of Animal Science and Veterinary Medicine, Shandong Agricultural University, Taian 271018 Shandong Province, P.R. China; 2 College of Life Science, Linyi University, Linyi, 276005, China

## Abstract

**Background:**

MicroRNAs have been suggested to play important roles in the regulation of gene expression in various biological processes. To investigate the function of miRNAs in chicken ovarian development and folliculogenesis, two small RNA libraries constructed from sexually mature (162-day old) and immature (42-day old) ovary tissues of Single Comb White Leghorn chicken were sequenced using Illumina small RNA deep sequencing.

**Results:**

In the present study, 14,545,100 and 14,774,864 clean reads were obtained from sexually mature (162-d) and sexually immature (42-d) ovaries, respectively. In total, 202 known miRNAs were identified, and 93 of them were found to be significantly differentially expressed: 42 miRNAs were up-regulated and 51 miRNAs were down-regulated in the mature ovary compared to the immature ovary. Among the up-regulated miRNAs, gga-miR-1a has the largest fold-change (6.405-fold), while gga-miR-375 has the largest fold-change (11.345-fold) among the down-regulated miRNAs. The three most abundant miRNAs in the chicken ovary are gga-miR-10a, gga-let-7 and gga-miR-21. Five differentially expressed miRNAs (gga-miR-1a, 21, 26a, 137 and 375) were validated by real-time quantitative RT-PCR (qRT-PCR). Furthermore, the expression patterns of the five miRNAs were analyzed in different developmental stages of chicken ovary and follicles of various sizes.

**Conclusion:**

The present study provides the first miRNA profile in sexually immature and mature chicken ovaries. Some miRNAs such as gga-miR-1a and gga-miR-21are expressed differentially in immature and mature chicken ovaries as well as among different sized follicles, suggesting an important role in the follicular growth or ovulation mechanism in the chicken.

## Background

In chicken, ovarian function determines laying performance. It has been estimated that approximately 12,000 oocytes are present in the hen ovary when sexually mature, but only a few hundred oocytes are selected to reach maturity and ovulate. Usually, the functionally mature hen ovary contains hundreds of white cortical follicles with 1–5 mm in diameter, 5–6 small yellow pre-hierarchical follicles with 6–8 mm in diameter and 5–6 large yellow preovulatory hierarchy follicles with 9–40 mm in diameter [1]. In the hen ovary, a single follicle is selected into the preovulatory hierarchy from a small cohort of pre-hierarchical follicles approximately once a day [2]. The preovulatory follicles are arranged in a hierarchical order in which the largest one (F1) represents the most mature and will ovulate for egg formation first. Ovary development and folliculogenesis undergo a series of complex processes including modulation of gonadotropin action, steroid hormones biosynthesis, follicle selection, granulosa cell proliferation and differentiation, which require tightly regulated expression and interaction of a multitude of genes at the transcriptional and post-transcriptional levels [3-7].

MicroRNAs (miRNAs) are a class of endogenous non-coding small regulatory RNAs of 19–24 nucleotides in size [8,9] that can regulate gene expression by targeting specific sites in the 3′-untranslated region (3′-UTR) of mRNAs [10-13]. The seed region, which is located at miRNA nucleotides 2–8 from the 5′ end of a miRNA sequence, is the most important sequence for interaction with mRNA targets [14,15]. Increasing evidence indicates that miRNAs play important roles in post-transcriptional regulation of gene expression and have been shown to control multiple biological and metabolic processes such as organogenesis, development, cell proliferation and differentiation as well as many diseases [16-20]. The mechanism of miRNA regulated gene expression is very complex because one miRNA can target several thousand mRNAs, and one mRNA can be regulated by several miRNAs [8,21]. Identifying the expression patterns of miRNAs in the hen ovary is the first step to elucidating their functions in ovarian development and folliculogenesis. Several studies have demonstrated that inactivation of Dicer1 can result in accelerated early follicle recruitment and degeneration as well as infertility and the dysfunction of oocyte maturation and ovulation [22-24]. Furthermore, genome-wide miRNA expression has been examined in the ovaries of mice [25,26], cattle [27-29], pig [30] and sheep [31]. These studies show that miRNAs play a critical role in the development of mammalian gonads, but little is known about the involvement of miRNAs in adult chicken ovary and follicle development. The aim of this study was to identify the differentially expressed miRNAs in sexually mature and immature chicken ovaries by using Illumina small RNA deep sequencing (Illumina Genome Analyzer). This approach is highly suited for small RNA discovery, which not only provides sequences of low abundance species but also provides quantitative data as the frequency of sequencing reads reflects the abundance of miRNAs in the population [32]. Finally, we validated and identified the expression pattern of gga-miR-1a, 21, 26a, 137 and 375 at different developmental stages of ovary and follicles of various sizes using qRT-PCR. These results may help us to further understand how ovarian function is regulated by miRNAs.

## Results

### Sequence analysis of small RNAs in chicken ovary

To survey miRNAs involved in the maturity of the chicken ovary, two small RNA libraries were generated from 42-d and 162-d chicken ovaries that, respectively, represent sexually immature and mature ovaries, and the libraries were sequenced by Illumina small RNA deep sequencing technology. In total, 16,817,204 and 17,936,768 raw reads were obtained from the sexually mature (162-d) and sexually immature (42-d) ovary libraries, respectively. After filtering the low-quality sequences, empty adaptors and single-read sequences, 14,545,100 and 14,774,864 clean reads of 18–30 nt were selected for further analysis from the sexually mature (162-d) and sexually immature (42-d) ovary libraries, respectively. Among the selected reads, 9,619,834 sequences from the mature ovary and 11,019,895 sequences from the immature ovary mapped perfectly to the chicken genome (Additional file 1: Table S1), amounting to 66.14 and 74.59% of the total reads, respectively; and 5,834,400 reads in mature and 3,919,973 reads in immature ovaries were found to be similar to miRNAs. The rest of the sequences were found to be other types of RNA, including noncoding RNA, tRNA, rRNA, snRNA or snoRNA. The number and proportions of the categories of small RNAs found are given in Additional file 1: Table S1.

**Figure 1:**
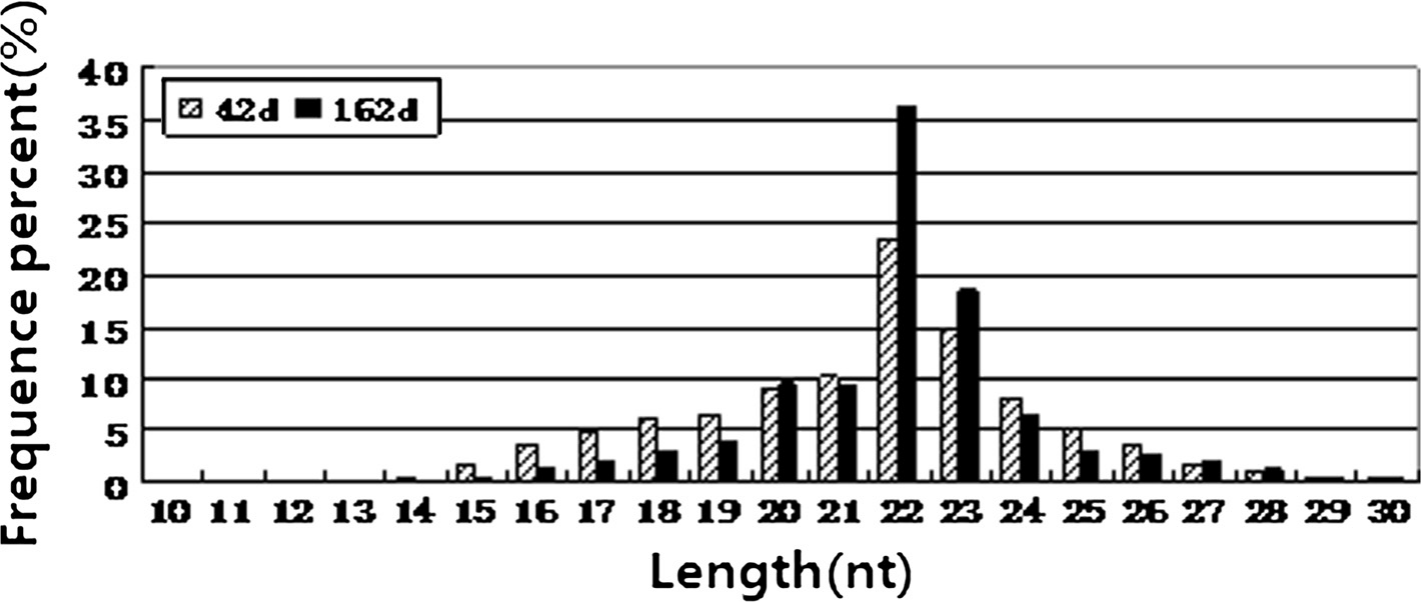
**Length distribution and abundance of small RNAs sequences in chicken ovary by Illumina small RNA deep sequencing.** Sequence length distribution of clean reads based on the abundance and distinct sequences; the most abundant size class was 22 nt, followed by 23 nt and 21 nt.

The size distribution of small RNAs (sRNAs) was similar in the two libraries, and the majority of them were from 20 to 24 nt (Figure 1). The most abundant size class was 22 nt, which accounted for 36.12% and 23.58% of the total reads in sexually mature and immature chicken ovaries, respectively, followed by 23 nt (18.39%, 14.54%) and 21 nt (9.39%, 10.2%).

### Identification of known miRNAs in chicken ovary

To investigate the expression of known miRNAs in the chicken ovary, small RNA sequences identified by Illumina small RNA deep sequencing were compared with known mature miRNAs and precursors in miRBase (version 14, 2009 and version 19, 2012). The results showed that 232 mature miRNA, 35 miRNA*, 260 miRNA-5p and 3396 miRNA-3p were found in the two libraries, and 202 known miRNAs are co-expressed in the two libraries (Additional file 1: Table S2).

The Illumina small RNA deep sequencing approach allows us to determine the relative abundance of various miRNA families by calculating the sequencing frequency. A highly expressed miRNA will likely have a large number of sequenced clones [33]. In the mature and immature chicken ovary libraries, known miRNAs had a broad range of expression levels; some (such as miR-10a, 21 and 101) were found to have more than hundreds of thousands of sequence reads, while others (such as miR-124a, 3540 and 1759) had less than 20 (Additional file 1: Table S2), indicating that expression varies significantly among different miRNA families. The proportion of different categories of small RNAs often reflects the roles in a particular tissue or different developmental stages and their associated biological mechanisms. The following 15 miRNAs were dominantly expressed in the two libraries: gga-miR-10a, gga-miR-146c, gga-miR-101, gga-miR-21, gga-let-7a, gga-let-7b, gga-let-7c, gga-let-7j, gga-let-7f, gga-let-7 k, gga-miR-30a-5p, gga-miR-30e, gga-miR-148a, gga-miR-100 and gga-miR-126. Each of these 15 miRNAs had more than 100,000 reads, while the majority of other miRNAs sequenced had more than 500 reads. The most abundant miRNA in the mature ovary was gga-miR-10a, which had 1,177,256 reads, followed by gga-miR-21, which had 929,545 reads. However, in the immature ovary, gga-let-7a (415,122 reads) and gga-let-7j (413,833 reads) were the most abundant (Additional file 1: Table S2). Sequence analysis indicated that the relative abundance of members within one miRNA family varied greatly in the chicken ovary, suggesting functional divergence within the family. For example, in the immature ovary, gga-let-7 family abundance varied from 855 reads (gga-let-7d) to 415,122 reads (gga-let-7a). Similarly, other miRNA families exhibited vast read ranges, such as the gga-miR146 (91–235,844 reads) and gga-miR-30 (886–164,361 reads) families (Additional file 1: Table S2). These results indicate that different members within one miRNA family can have clearly different expression levels, most likely due to tissue- or developmental stage-specific expression.

In the two developmental stages of the chicken ovary, a total of 19 miRNA*s were co-expressed (Additional file 1: Table S3). It has been demonstrated that the miRNA* is generally less stable than their mature miRNA counterparts. During the biogenesis of the mature miRNAs, some of the miRNA* strands are degraded rapidly [34,35]. Generally, the miRNA* has lower expression levels than their corresponding mature miRNAs. In our data, the majority of miRNA*s had much fewer reads than their corresponding mature miRNAs. One intriguing exception was gga-miR-140*, which was present as 105,474 reads in the mature ovary and 95,992 reads in the immature ovary; however, gga-miR-140 had only 7,370 and 7,953 reads in each library, respectively. Similar expression patterns were found for gga-miR-199* and gga-miR-202* (Additional file 1: Table S3). Another interesting case was gga-miR-551*, in which only miRNA* was detected. These results suggest that miRNA*s may play some functional roles during chicken ovary development.

### miRNAs differentially expressed in sexually mature and immature chicken ovaries

**Figure 2:**
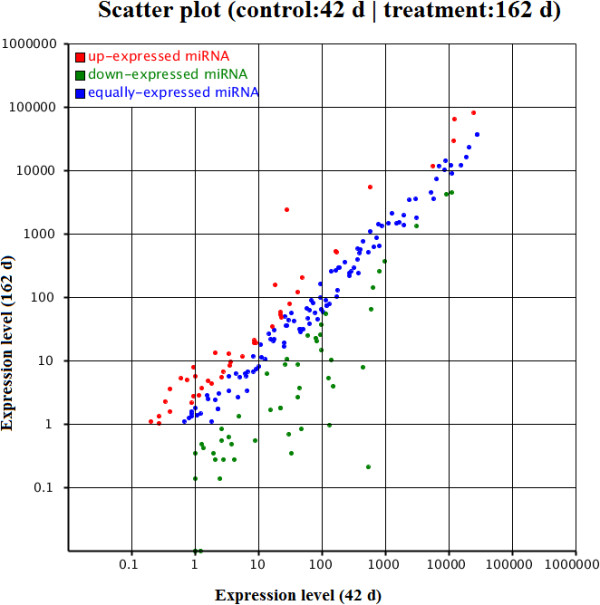
**Comparison of expression levels of miRNAs in 42-d and 162-d ovary.** The X and Y axes show expression levels of miRNAs in each library. Red points represent up-expressed miRNAs; green points represent down-expressed miRNAs; blue points represent equally-expressed miRNAs; the expression level in 42-d was as the control.

The main purpose of the study was to identify miRNAs involved in chicken ovary and follicle development. According to the changes in relative miRNA abundance between the two libraries, 93 miRNAs were expressed significantly differently between sexually mature and immature ovaries (Additional file 1: Table S4). In comparison with the sexually immature ovary (42-d), 42 miRNAs were significantly up-regulated, and 51 miRNAs were significantly down-regulated in the sexually mature ovary (162-d) (|log2Ratio| ≥ 1, P-value ≤ 0.05) (Additional file 1: Table S4). The majority of differentially expressed miRNAs range from a 2- to 4-fold difference, and only 15 miRNAs showed differences greater than 4-fold between the two libraries (Figure 2 and Table 1). Among the up-regulated miRNAs, gga-miR-1a had the highest fold-change with 6.405-fold. Among the down-regulated miRNAs, gga-miR-375 had the highest fold-change with 11.345-fold, followed by gga-miR-217, gga-miR-458b, gga-miR-449c and gga-miR-124a with more than 6-fold (Additional file 1: Table S4). In addition, we found that two members of the let-7 miRNA family, gga-let-7b and gga-let-7 g, exhibited significantly differential expression, although the fold-changes were lower at 1.292- and 1.366-fold, respectively.

**Table 1:** Fifteen differentially expressed miRNAs regulated greater than 4-fold in chicken mature and immature ovary (162d/42d)

**miR-name**	**Fold-change**	**P-value**	**regulated**	**Sig-lable**
gga-miR-375	11.345	0	down	**
gga-miR-217	7.1082	0	down	**
gga-miR-458b-5p	6.9287	4.42E-06	down	**
gga-miR-449c-5p	6.6656	3.46E-05	down	**
gga-miR-124a	6.6656	3.46E-05	down	**
gga-miR-216a	6.5919	1.08E-136	down	**
gga-miR-10a	6.4055	0	up	**
gga-miR-34b	5.8478	8.82E-188	down	**
gga-miR-7	5.8327	0	down	**
gga-miR-216b	5.4757	3.08E-188	down	**
gga-miR-34c	5.2853	0	down	**
gga-miR-137	4.5848	0	down	**
gga-miR-1720-3p	4.1869	1.98E-09	down	**
gga-miR-9-3p	4.0218	6.41E-30	down	**
gga-miR-135a	4.0032	1.33E-134	down	**

** represents fold change (log2)>1 or fold change(log2)<-1, and *P*-value<0.01.

**Figure 3:**
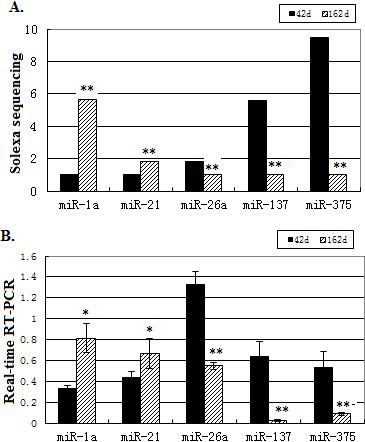
**qRT-PCR validation of five differentially expressed miRNAs identified using Illumina small RNA deep sequencing. *A****.* Fold-change of five miRNAs that were differentially expressed between 42-d and 162-d ovaries based on deep sequencing data. ***B****.* The relative expression abundance of the five miRNAs between 42-d and 162- d ovaries by real-time quantitative RT-PCR. * *P* < 0.05,** *P* < 0.01.

To validate the Illumina small RNA deep sequencing data, five differentially expressed miRNAs (gga-miR-1a, gga-miR-21, gga-miR-26a, gga-miR-137 and gga-miR-375) were selected, and their expression levels were quantified using real-time quantitative RT-PCR (qRT-PCR). The results were consistent with the deep sequencing data (Figure 3). These results provide evidence that Illumina small RNA deep sequencing is a sensitive and reliable approach to identifying differentially expressed miRNAs in the chicken ovary.

### Developmental expression patterns of five miRNAs in the ovary and follicles

To further characterize the functionality of these differentially expressed miRNAs identified from the chicken ovary, the expression levels of gga-miR-1a, gga-miR-21, gga-miR-26a, gga-miR-137 and gga-miR-375 were further examined in ovary tissues from 42-, 70-, 90-, 110- and 162-day-old White Leghorn hens (n =3), as well as in follicles isolated from ovaries of 162-day-old White Leghorn hens, namely, a large white follicle (LW, diameter =2-4 mm), small yellow follicle (SF, diameter =6-8 mm), F6 (diameter =12-14 mm), F4 (diameter =22-24 mm), F2 (diameter =30-31 mm) and F1 (diameter =34 mm) follicles.

**Figure 4:**
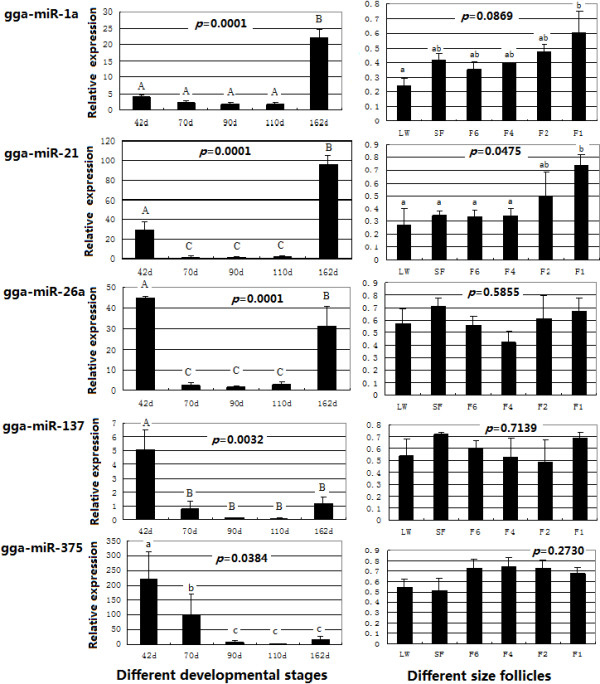
**Expression patterns of gga-miR-1a, gga-miR-21, gga-miR-26a, gga-miR-137 and gga-miR-375 in different developmental stages of ovary and in different sized follicle in chicken by qRT-PCR assays.** The mean expression value is calculated using 5S rRNA as control, according to the ^△△^Ct method. Error bars represent one standard deviation of three different biological replicates. Statistical significance is reported for each miRNA. a, b and c: *P* < 0.05; A, B and C: *P* < 0.01.

The results showed that some of the miRNAs exhibit developmental stage-specific expression patterns in ovarian development (Figure 4). The expression patterns of gga-miR-1a and gga-miR-21 were similar in the different developmental stages; relatively lower expression was observed from 42-d to 110-d compared with 162-d and increased dramatically to peak in 162-d. However, the expression dynamics of gga-miR-26a were different; the highest expression level was found in the 42-d ovary, then decreased from 70-d to 110-d, and finally increased in the 162-d ovary although still lower than 42-d. The expression of gga-miR-137 and gga-miR-375 in ovary decreased significantly from 42-d to 162-d.

In follicles (Figure 4), the expression patterns of gga-miR-1a and gga-miR-21 were similar; during the development from LW to F1, the expression levels of the two miRNAs increased progressively with F1 having the highest level, which suggests that gga-miR-1a and gga-miR-21 may be involved in the follicular growth or ovulation mechanism in the chicken. For gga-miR-26a and gga-miR-137, the highest expression level was found in the SF (6–8 mm) follicle. The expression of gga-miR-375 was relatively lower in LW and SF follicles, increased most in the F6-F2 follicles and then declined in the F1 stage, suggesting an important role of gga-miR-26a and gga-miR-137 in hierarchy maintenance of follicles in chicken.

### Target prediction and function annotation

**Figure 5:**
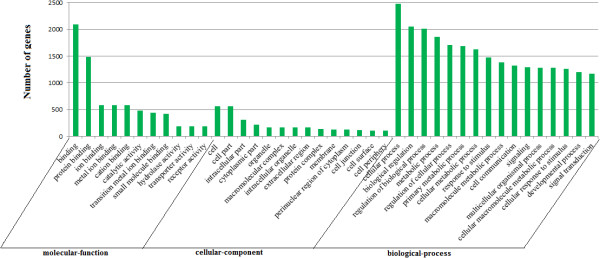
**Partial gene ontology (GO) classification annotated for predicted target genes of 93 differentially expressed miRNAs.** The figure shows partial GO enrichment for the predicted target genes in molecular function, cellular component and biological processes.

To further understand the physiological functions and biology processes involved by 93 differentially expressed miRNAs during ovary development, target prediction was performed by integrating three public databases (TargetScan, PicTar and miRanda), and 3,122 target genes were identified (data not shown). GO annotation and KEGG pathway analysis were performed to identify functional modules regulated by these miRNAs. The GO annotation enrichment results showed that regulation of biological process, cellular metabolic process, developmental process and signal transduction are the most significantly enriched GO terms (Figure 5). The KEGG pathway analysis revealed 178 pathways that were enriched with miRNA targets (data not shown). MAPK signaling pathway, Calcium signaling pathway, Wnt-signaling pathway Insulin signaling pathway and GnRH signaling pathway ranked among the most enriched pathways (Table 2). Although the false-positive predictions always exist, we suggest that these targets have high possibility of being regulated by miRNAs in chicken ovary development.

**Table 2:** The most enriched KEGG pathways of target genes for differentially expressed miRNAs

**Pathway**	**Genes count**	**P-value**	**Pathway ID**
MAPK signaling pathway	320	0.8154	ko04010
Calcium signaling pathway	264	0.8454	ko04020
ECM-receptor interaction	210	0.9928	ko04512
Axon guidance	210	0.8752	ko04360
Wnt signaling pathway	209	0.8757	ko04310
Insulin signaling pathway	191	0.8859	ko04910
Oocyte meiosis	146	0.9117	ko04114
TGF-beta signaling pathway	123	0.9251	ko04350
Gap junction	122	0.9257	ko04540
GnRH signaling pathway	121	0.9263	ko04912
PPAR signaling pathway	108	0.9340	ko03320
Progesterone-mediated oocyte maturation	108	0.9340	ko04914
p53 signaling pathway	97	0.9405	ko04115
VEGF signaling pathway	86	0.9471	ko04370

## Discussion

The Illumina deep sequencing platform is efficient for miRNA discovery and is widely used to generate small RNA profiles in various organisms. In the present study, we obtained detailed miRNA profiles of sexually mature (162-d) and immature (42-d) chicken ovaries using this method. To our knowledge, this is the first report of miRNA expression profiles of sexually immature and mature ovaries in the chicken. The sequence analysis showed that the dominant size of small RNAs in chicken ovary was 22 nt followed by 23 and 21 nt sequences. This result is typical of Dicer-processed small RNA products and was consistent with the known 19–24 nt range for miRNAs. Our data are consistent with previous findings in mouse [25] and pig [30] testis and ovary as well as chicken skeletal muscle [36] and somites [37]. However, in Holstein Cattle testis and ovary, the 20 nt size is the most abundant, followed by 22 nt [29], while another study in bovine ovary indicated that 21 nt is the predominant size [27]. This is likely caused by difference in species or developmental stages.

In the sexually mature chicken ovary library, gga-miR-10a and gga-miR-21 were the two most frequently sequenced miRNAs, and the let-7 miRNA family was another abundant cluster with let-7a being the most abundantly expressed miRNA. The let-7 miRNA family was also expressed abundantly in ovary and oocyte of bovines [28,29,38,39], as well as in murine ovaries and testis [40]. Furthermore, gga-miR-101, gga-miR-1a, gga-miR-146c, gga-miR-148a, gga-miR-126, gga-miR-26a and gga-miR-30d were abundant in our sequencing libraries, as has been shown in other animal gonads [25,27,28]. These miRNAs may have important roles in female reproductive physiology. A previous study reported that miR-21 was significantly up-regulated in murine granulosa cells before and 4 h after the hCG/LH surge and that it plays a role in preventing apoptosis in periovulatory granulosa cells as they transit to luteal cells. Knockdown of miR-21 in granulosa cells resulted in increased apoptosis and was associated with a reduced ovulation rate [41,42]. Some studies have shown that miR-21 was down-regulated by estradiol in MCF7 cancer cells [43,44]. In the present study, gga-miR-21 exhibited a significant 2.39-fold increase in a sexually mature ovary compared with an immature ovary, and the highest level was in F1 follicles, implying that gga-miR-21 plays an important role in sexual maturation in hens.

In this study, gga-let-7b is up-regulated while gga-let-7 g, gga-miR-17-3p gga-miR-30d, gga-miR-29a, gga-miR-26a and gga-miR-181a are all down-regulated in the mature ovary compared with the immature ovary. It has also been reported that miR-30c, let-7a, let-7b and let-7c were up-regulated, while miR-30d, miR-29a and miR-26a were down-regulated in rat granulosa cells treated with FSH for 12 h, which suggests that these miRNAs participate in FSH-mediated progesterone biosynthesis of granulosa cells [45]. Another study showed that a lack of miR17-5p and let-7b resulted in corpus luteum insufficiency and infertility in mice and that exogenous administration of the two miRNAs could prevent this phenotype [46]. A recent study showed that the activated estrogen receptor can suppress the expression of BAX by up-regulating a group of miRNAs including hsa-let-7 family members in endometrial adenocarcinoma and precancerous lesions [47]. In the same study, miR-181a and miR-30d are significantly down-regulated after estradiol treatment.

miR-26a is reported to have anti-apoptotic effects in many cancers [48-51]. In MCF7 cancer cells, estradiol can repress miR-26a expression to regulate numerous genes associated with cell growth and proliferation [44]. miR-26a was also found to play a role in normal tissue growth and development and to have an impact on cell proliferation and differentiation. One study showed that, during myogenesis, overexpression of miR-26a targets the enhancer of Zeste homolog 2 (Ezh2), which normally suppresses skeletal muscle differentiation [52]. In osteogenesis, miR-26a was found to regulate osteoblast cell growth and differentiation in human adipose tissue-derived stem cells [53]. In this study, the highest level of gga-miR-26a was found in SF follicles. SF were to be chosen from pre-hierarchical follicles (<8 mm) to pre-ovulatory hierarchy follicles (>10 mm), therefore, gga-miR-26a is likely associated with the mechanism of recruitment of dominant follicle in chicken.

Studies indicated that gga-miR-31, gga-miR-101, gga-miR-202 and gga-miR-202* may be involved in regulating gonadal differentiation in embryonic chicken gonads [54-56]. Reduced miR-202* expression is correlated with reduced expression of the testis-associated genes DMRT1 and SOX9, and up-regulation of the ovary-associated genes FOXL2 and CYP19A1 (aromatase) [55]. FOXL2 is a critical factor required for ovarian growth and differentiation whose functions include regulating aromatase enzyme expression, inhibins and follistatin gene expression, and granulosa cell development [57,58]. In our study, gga-miR-202* was down-regulated by 3.2-fold and gga-miR-31 was down-regulated by 1.68-fold in sexually mature ovaries compared with immature ovaries, suggesting they are also involved in the sexual maturity of ovary.

Bioinformatic analysis shows that miR-101 targets TGIF1 (TGFB-induced factor homeobox 1), ZEB2 (zinc finger E-box binding homeobox 2) and BMPR1B, which participate in the regulation of TGF-β signaling [56]. TGF-β signaling is critical to folliculogenesis and oogenesis in mammalian ovaries [59]. The increase in female miR-101 expression in differentiating ovaries can ease repression of TGF-β signaling [60]. In this study, gga-miR-101 was abundantly expression in chicken ovary and 123 putative target genes were involved in TGF-β signaling pathway. Study has shown that overexpression of miR-375 suppressed glucose-induced insulin secretion, and conversely, inhibition of endogenous miR-375 function enhanced insulin secretion [61]. In this study, gga-miR-375 is the largest down-regulated by 11-fold in the mature ovary and 191 putative target genes were involved in insulin signaling pathway.

## Conclusion

The present study is the first to examine the miRNA expression profile of sexually mature and immature ovaries in chicken, and evaluate miRNA function during ovary development and folliculogenesis. We identified 93 known miRNAs that were differentially expressed in mature and immature chicken ovaries that could exert novel functions in the regulation of ovarian development and folliculogenesis. Some miRNAs such as gga-miR-1a and gga-miR-21are expressed differentially in immature and mature chicken ovaries as well as among different sized follicles, suggesting an important role in the follicular growth or ovulation mechanism in the chicken. Further investigation concerning the function of these miRNAs should facilitate our understanding of the regulatory roles of miRNAs in regulating chicken ovary and follicle growth. The role of individual miRNAs in chicken ovarian development and follicle growth requires further investigation.

## Methods

### Collection of chicken ovary and follicle tissues

A total of 30 one-day-old female Single Comb White Leghorn chickens (*Gallus gallus domesticus*) were obtained from Shanghai Poultry Breeding Co Ltd and were reared in the brooding temperature at 35°C (65% RH) for the first 5 days. The lighting program was 23 h light: 1 h darkness. The chickens were reared under natural temperature and light (latitude: 36°28′ N; longitude: 117°59′; June to October, 2009). All chickens received a starter diet with 19% crude protein and 11.97 MJ/kg of metabolizable energy; after day 42 they received a grower diet with 16.5% crude protein and 11.97 MJ/kg of metabolizable energy. Feed and water were freely available during the rearing period.

Samples were collected when the chickens were 42, 70, 90, 110 and 162 days-old. At each stage, three chickens were randomly slaughtered and ovaries were collected, and the following follicles were collected from 162-d ovary: F1 (34 mm), F2 (30–31 mm), F4 (22–24 mm), F6 (12–14 mm), small yellow follicle (SF, 6–8 mm) and large white follicle (LW, 2–4 mm). Further, three whole ovaries from 42- and 162-day-old Single Comb White Leghorn hen were collected separately to prepare two pools representing sexually immature and sexually mature ovaries for constructing small RNA libraries. These tissues were frozen in liquid nitrogen for RNA isolation.

All animal experiments were approved by the Institutional Animal Care and Use Ethics Committee of Shandong Agricultural University and performed in accordance with the “Guidelines for Experimental Animals” of the Ministry of Science and Technology (Beijing, China).

### Small RNA library construction and Illumina small RNA deep sequencing

Two small RNA libraries pooled from immature ovaries (42-d) and mature ovaries (162-d) were constructed. Total RNA was extracted using Trizol (Invitrogen) according to the manufacturer’s protocol, and the quantity of RNA was examined by using an Agilent 2100 Bioanalyzer. From each sample, 20 μg of total RNA was used for Solexa sequencing by an Illumina Genome Analyzer (Illumina, San Diego, CA, USA) at the Beijing Genomics Institute (BGI) (Shenzhen, Guangzhou, China). Briefly, the 16–30 nt fraction of total RNA was purified and enriched using denaturing polyacrylamide gel electrophoresis (PAGE). Then 3′ and 5′ RNA adapters were each ligated with T4 RNA ligase. Subsequently, the small RNAs ligated to adapters were subjected to RT-PCR amplification for 15 PCR cycles. The amplification products (70–90 bases in length, small RNA and adapters) were further purified on a 6% polyacrylamide TBE gel and used for sequencing analysis. Sequencing reads were extracted from the image files generated by the Illumina/Solexa 1G Genome Analyzer.

### Bioinformatic analysis of sequencing data

After filtering adaptor sequences and removal of contaminated reads, the clean reads were processed for computational analysis. First, the clean reads were mapped to the UCSC chicken genome galGal3 using NCBI MegaBLAST and rRNA, tRNA, snRNA, scRNA and snoRNA were discarded from the small RNA sequences. Subsequently, the remaining sequences were analyzed by a BLAST search against the miRNA database, miRBase (version14.0 and 19.0) [62], to identify the conserved miRNAs in *Gallus gallus*. Only the perfectly matched sequences were considered to be conserved miRNAs. To analyze differential miRNA, miRNAs expression in each library (42-d and 162-d chicken ovary) was normalized to obtain the expression of transcripts per million using the following formula: Normalized expression = (Actual miRNA sequencing reads count / Total clean reads count) × 1,000,000. If the normalized expression (NE) value of a given miRNA is zero, the expression value was modified to 0.01. If the normalized expression of a given miRNA is less than 1 in both libraries, it was removed in future differential expression analyses. The fold-change and P-value were calculated from the normalized expression. When |log2Ratio| ≥ 1 and P-value ≤ 0.05, it was be seen as differential expression [63].

### Quantitative real-time PCR of miRNAs

To validate and characterize the differentially expressed miRNAs identified using high-throughput sequencing, five miRNAs were selected, and we analyzed their relative expression levels in ovaries at 42, 70, 90, 110 and 162 days of age as well as in different sized follicles. Real-time quantitative PCR was performed using Mx3000p^™^ SYBR® Green Real-time quantitative PCR Analyzer (Stratagene, USA). Briefly, 3 μg miRNA was reverse transcribed to cDNA using One Step PrimeScript® miRNA cDNA Synthesis Kit (Tiangen Biotech Co., China). The reverse transcriptase reaction consisted of 10 μL 2× miRNA Reaction Buffer Mix, 2 μL 0.1% BSA, 2 μL miRNA PrimeScript® RT Enzyme Mix, 2 μL total RNA (2 μg/μL) and RNase-free dH_2_O up to 20 μL. The RT-PCR program was 37°C for 60 min and 85°C for 5 sec. The cDNA products were stored at −20°C. Real-time quantitative PCR was performed with SYBR® Premix Ex Taq^™^ II (Tiangen Biotech Co., China). The reaction solution was prepared on ice, and the components were 10 μL SYBR® Premix Ex Taq^™^ II (2×), 0.8 μL PCR Forward Primer (10 μM), 0.8 μL Uni-miR qPCR Primer (10 μM), 0.4 μL ROX Reference Dye II(50×), 3 μL cDNA, and dH_2_O up to 20 μL. The reaction mixtures were incubated in a 96-well plate at 95°C for 30 sec followed by 40 cycles of 95°C for 5 sec, 60°C for 30 sec and 72°C for 30 sec. All reactions were run in triplicate. The primers for miRNAs have the same sequences as *Gallus gallus* miRNAs with an appropriate adjustment at their 5′ terminus. The relative expression quantification was calculated using the 2^−ΔΔCt^ method after the threshold cycle (*Ct*) and was normalized with the *Ct* of chicken 5S rRNA. Data are from three individuals. The each miRNA expression level was presented as 2^−ΔΔCt^ means ± SE (standard error), and error bars indicate the standard error of 2^−ΔΔCt^ mean values. One-way ANOVA and Duncan’s Multiple Range test (*P* < 0.05) (SAS version 8.02, 2001) were used to examine the significance of differential expression level in each miRNA between different stages ovaries or different size follicles, and the difference was considered as significant when *P* <0.05.

## Competing interests

The authors declare that they have no competing interests.

## Authors’ contributions

LK designed and performed the experiments and drafted the manuscript. XC carried out the qRT-PCR. YZ and CY carried out animal care and prepared samples. YJ conceived the study and the experimental design and helped draft the manuscript. All authors read and approved the final manuscript.

## Supplementary Material

Additional file 1: Table S1 Summary of Illumina small RNA deep sequencing data for small RNAs in mature and immature chicken ovaries. **Table S2.** The expression patterns of known miRNAs in the two libraries of chicken ovary. **Table S3.** The expression patterns of miRNA*s and their corresponding miRNAs in chicken ovary. **Table S4.** The significantly differentially expressed known miRNAs in chicken ovary. Click here for file
